# The organizational side of a disruption mitigation process: exploring a case study during the COVID-19 pandemic

**DOI:** 10.1007/s12063-022-00264-w

**Published:** 2022-04-26

**Authors:** Margherita Molinaro, Pietro Romano, Gianluca Sperone

**Affiliations:** 1 Polytechnic Department of Engineering and Architecture, University of Udine, Udine, Italy; 2 Zoppas Industries Heating Element Technologies, Vittorio Veneto, Treviso, Italy

**Keywords:** Supply chain disruptions, Reactive organizational practices, Mitigation process, COVID-19 pandemic

## Abstract

This paper deals with the mitigation process of the COVID-19 pandemic. Scholars propose and discuss several mitigation strategies to face the COVID-19 disruptions, mainly focusing on technology and supply chain redesign related aspects. Less attention has been paid to the organizational aspects of the mitigation process. We address this gap through an in-depth analysis of the reactive organizational practices implemented by an Italian company during the COVID-19 pandemic. We further compare these practices with those proposed in the disruption management literature to identify common traits and differences. The results show that the overall management of a pandemic’s mitigation process does not significantly differ from that of conventional disruptions, since both contexts require the same basic organizational practices. However, some peculiarities on how these practices should be implemented in a pandemic setting do emerge, such as the implementation of a cyclic rather than linear problem-solving process, the adoption of a learning-by-doing approach, the need of a risk-taker mindset and the importance of creativity and improvisation. Besides complementing the literature, these findings allow to provide indications to managers on how to organize and coordinate the activities during the mitigation process, as well as on what capabilities and competencies should be leveraged to face the pandemic’s disruptions.

## Introduction

Since more than one year, the Corona Virus Disease 2019 (COVID-19) has been causing an unprecedented series of supply chain shocks that go beyond the more traditional disruptive threats and events (Belhadi et al. 2021; Nikolopoulos et al. 2021; Qin et al. 2021). According to many, this pandemic is unique. On the one hand, it is characterized by the three main peculiarities of an epidemic outbreak, namely (1) long-term disruption with unexpected scaling; (2) simultaneous disruption propagation in both supply chains and population; (3) simultaneous disruption on supply, demand and logistics (Ivanov 2020). On the other hand, compared to recent epidemic outbreaks such as Ebola, Swine flu or SARS, it is more pervasive in spread and can thus be considered one of the most severe disruptive events in recent history (Ivanov and Dolgui 2020; Okorie et al. 2020). Therefore, a thorough investigation of COVID-19 impacts, consequences and countermeasures becomes fundamental, not only to better understand its features, but also to identify guidelines for any future comparable situations (Singh et al. 2020).

Not by chance, the COVID-19 literature is already quite developed. Some papers investigate the issues and challenges faced by companies during the pandemic, which include supply shortages, demand uncertainty, transportation issues and carriers’ cost increase (Sharma et al. 2020a, 2020b; van Hoek 2020; Finkenstadt and Handfield 2021; Magableh 2021). Other scholars look at new or alternative solutions to carry out the supply chain processes during a pandemic, such as planning, forecasting and demand management (see Govindan et al. 2020; Nikolopoulos et al. 2021) or waste management (see Lotfi et al. 2021; Tirkolaee et al. 2021), while others discuss the tendencies that will characterize the aftermath of COVID-19 (e.g., de Sousa Jabbour et al. 2020; Sarkis 2020). Indeed, the most investigated research stream includes papers focusing on the mitigation strategies adopted by companies to deal with the pandemic effects, including supply chain localization, technology exploitation, inventories and reserve capacity (e.g., Paul and Chowdhury 2020; van Hoek 2020; Butt 2021). A deep exploration and understanding of mitigation strategies and reactive practices is considered increasingly important, given the large magnitude and unexpected evolution of recent extreme disruptions (Chen et al. 2019).

However, looking at COVID-19 literature, we can observe that the mitigation strategies explored and discussed by scholars include solutions mainly linked to technology and supply chain structure, such as the identification of new or alternative suppliers (e.g., van Hoek 2020), the increase of production capacity (e.g., Paul and Chowdhury 2020) or the implementation of technological solutions to improve visibility along the network (e.g., Sharma et al. 2020a). What actually lacks is a focus on how to manage the organizational aspects of the mitigation, in terms of organizational structure and culture, roles and responsibilities, procedures and coordination mechanisms, steps and sequence of the mitigation process. Although these aspects have been deeply investigated in the disruption management literature (e.g., Macdonald and Corsi 2013; Dabhilkar et al. 2016), they are still overlooked by COVID-19 related studies and no shared guidelines exist on how companies should organize the mitigation process in the COVID-19 pandemic setting. As we previously highlighted, a pandemic is a unique event and the conventional strategies to organize the mitigation process may not be equally effective in such a context, as suggested by DuHadway et al. (2019).

This paper seeks to address the mentioned gap by exploring the reactive organizational practices to manage the mitigation process in the context of COVID-19 pandemic. In addressing this gap, we also answer the call of van Hoek (2020) for a more empirical and event-based research on supply chain disruptions and resilience. In particular, the following two research questions guide our analysis:

RQ1. How does the overall management of a pandemic’s mitigation process differ from that of more conventional disruptions?

RQ2. What characterizes the effective reactive organizational practices to face an extremely disruptive event such as a worldwide pandemic?

To answer these questions, we thoroughly explore the case of an Italian company that, thanks to a structured organizational mitigation process, was able to avoid production interruptions and minimize the negative effects of the COVID-19 disruptions, despite the numerous challenges it had to face in both upstream and downstream networks.

The results of the case study show that the overall management of a pandemic’s mitigation process does not significantly differ from that proposed in general disruption studies. However, some peculiarities on how the organizational practices should be implemented during a pandemic emerge. On the one hand, these findings allow to complement the literature on COVID-19 related research and to specify the extent to which disruption management literature is applicable to a pandemic setting. On the other hand, they also build the basis to provide managerial indications on how to manage the organizational aspects of a pandemic’s mitigation process.

The paper has the following structure. In Sect. 2, we provide a literature review on general disruption management and on the stream specifically related to COVID-19, which ends with the identification of the research gap. In the following two sections, we first present the research methodology and then describe the case study. Section 5 analyzes the case study results, while Sect. 6 discusses them in light of existing literature, underlining theoretical and managerial implications. Finally, the last section provides some concluding remarks and reflections on research limitations and future research directions.

## Literature review

### Reactive organizational practices for disruption management

After the numerous disruptions that recently affected many supply chains all over the world, the interest of academics and practitioners in developing solutions to cope with such events has grown over the years (Li et al. 2020). In this context, several organizational practices have been proposed and discussed by scholars to manage the mitigation process of a disruption.

There’s sufficient convergence that the ability to effectively respond to a disruptive event firstly depends on a proper internal organization, based on cooperation and coordination and supported by a well-defined mitigation process. In this regard, Norrman and Jansson (2004) describe how Ericsson revised its risk management strategies after an incident at a sub-supplier caused a long production interruption. Besides a series of proactive activities, the company clearly established how to handle the post-disruption phase, introducing detailed response, recovery and restoration plans, assigning precise roles and responsibilities and establishing coordination-based procedures. More recently, Macdonald and Corsi (2013) interviewed several managers that experienced a disruption event and highlighted the importance of creating response teams to manage the mitigation process, involving members representing various roles within the focal company and, eventually, various parties of the supply chain. The size, experience and organization of this team significantly influence its effectiveness, together with the team leader(s)’ attributes. Furthermore, the team should receive not only enough space and resources, but also a proper top management support (Ponomarov and Holcomb 2009; Scholten et al. 2014). The use of standard procedures to ensure business continuity is a further requirement for an effective mitigation process, as highlighted by Chen et al. (2019) and Jüttner and Maklan (2011). According to many (e.g., Norrman and Jansson 2004; Tukamuhabwa et al. 2017), these procedures, together with all the activities needed in the mitigation process, should be included in a contingency plan, defined and organized in advance by the company. Jüttner and Maklan (2011) also claim the importance of internal coordination and cooperation, while Ellis et al. (2011) discuss the role of organizational aspects, such as work team composition, reward systems and culture, in supply disruption risk management.

Interpersonal relationships between supply chain partners are considered by many a significant antecedent of a firm’s resilience (Durach and Machuca 2018). Thus, besides internal practices, also external activities, based on frequent interactions with supply chain partners, are needed. A key role for an effective mitigation process is played by information sharing and dissemination along the supply chain (Craighead et al. 2007; Jüttner and Maklan 2011; Li et al. 2017), which should go beyond the direct network of the focal company to better evaluate the operational risk and should be accompanied by a structured collection of information from the focal company (Chen et al. 2019). The exchange speed and accuracy of this information plays a key role in affecting the mitigation process effectiveness (Chen et al. 2019). A detailed information management model to properly select, gather, process and share relevant data during a disruptive event is offered by Messina et al. (2020), who further highlight the importance of such activity. Finally, additional practices frequently mentioned in the literature are cooperation and coordination along the supply network, that allow to share the partners’ resources, align processes and activities and improve the effectiveness of the related results (Craighead et al. 2007; Chen et al. 2019; Messina et al. 2020).

After a detailed analysis of these practices, we classified them into three groups, as shown in Table 1: *governance*, which refers to organizational structure, culture and commitment; *interfaces*, which concern coordination and cooperation activities inside and outside companies’ boundaries; and *operations*, which is linked to the operational steps, procedures and practices of the mitigation process.

**Table 1:** Reactive organizational practices to manage traditional supply chain disruptions

Categories	Organizational practices	Exemplary references
*Governance*	Cross-functional task force creation	Macdonald and Corsi (2013); Scholten et al. (2014); Dabhilkar et al. (2016); Chen et al. (2019)
	Top management support and active involvement of managers	Ponomarov and Holcomb (2009); Scholten et al. (2014); Dabhilkar et al. (2016)
	Clear distribution of responsibilities	Norrman and Jansson (2004); Dabhilkar et al. (2016)
*Interfaces*	Cross-functional coordination and cooperation	Norrman and Jansson (2004); Jüttner and Maklan (2011); Scholten et al. (2014); Dabhilkar et al. (2016); Chen et al. (2019)
	Collaboration and information sharing with supply chain partners	Craighead et al. (2007); Jüttner and Maklan (2011); Dabhilkar et al. (2016); Tukamuhabwa et al. (2017); Chen et al. (2019); Messina et al. (2020)
*Operations*	Systematic problem-solving process implementation based on standard procedures	Norrman and Jansson (2004); Dabhilkar et al. (2016); Chen et al. (2019)
	Direct collection of relevant disruption information	Scholten et al. (2014); Dabhilkar et al. (2016); Chen et al. (2019)

### Mitigation strategies in the context of COVID-19 pandemic and research gap

The studies on the mitigation strategies adopted by companies to contrast the disruptive events caused by the COVID-19 pandemic are numerous. We provide below an overview of the strategies mostly mentioned in the literature, summarizing them in Table 2. Some of these strategies are particularly useful to mitigate the short-term consequences of the pandemic, while others can help to improve resilience in a longer-term perspective, as highlighted by Zhu et al. (2020).

**Table 2:** Mitigation strategies to manage supply chain disruptions caused by COVID-19 pandemic

Mitigation strategies	Exemplary references
Supply chain visibility and information sharing	Sharma et al. (2020a); van Hoek (2020); Belhadi et al. (2021); Butt (2021); Dohale et al. (2021); van Hoek (2021)
Supply chain collaboration	Sharma et al. (2020a); van Hoek (2020); Belhadi et al. (2021); Dohale et al. (2021); van Hoek (2021)
Inventories and reserve capacities	Belhadi et al. (2021); Butt (2021); Dohale et al. (2021); van Hoek (2021)
Production planning improvement	Butt (2021)
New, alternative or local suppliers’ identification	Sharma et al. (2020a); van Hoek (2020); Belhadi et al. (2021); Dohale et al. (2021); van Hoek (2021)
Suppliers’ monitoring	Butt (2021); Sharma (2020a)
Production capacity increase	Paul and Chowdhury (2020)
Alternative route options’ identification	Butt (2021)
Virtual marketplaces development	Belhadi et al (2021)
Postponement	Van Hoek (2020); Dohale et al. (2021)

One of the most mentioned mitigation strategies is the reliance on technological solutions to exploit various types of benefits. The managers interviewed by Sharma et al. (2020a) discuss the importance of enhancing supply chain visibility through the use of technology, while those involved by van Hoek (2020) recommend an acceleration of digitalization projects aimed at improving information sharing with partners, whose collaboration is considered fundamental by many (e.g., Sharma et al. 2020a; Belhadi et al. 2021). A review of planning parameters and the use of inventories and reserve capacity are strategies suggested instead by Belhadi et al. (2021) and Butt (2021), who also highlight the need to develop solutions to better support production planning. According to van Hoek (2020), however, companies should not rely too much on high inventory levels to mitigate the COVID-19 disruptions, since they cannot completely eliminate the problem of materials scarcity but only delay it. Further recurrent mitigation strategies are related to the supplier network, since, as highlighted by van Hoek (2021), procurement and supply management activities play a key role for the initial mitigation process of a pandemic. A restructuring of the network aimed at identifying new alternative or local suppliers is suggested by van Hoek (2020) and Belhadi et al. (2021), whereas Butt (2021) and Sharma (2020a) highlight the importance of monitoring suppliers’ inventory and production plans. Other proposed solutions to face the pandemic effects include increase of production capacity (Paul and Chowdhury 2020), development of virtual marketplaces (Belhadi et al. 2021), postponement to improve agility (van Hoek 2020), identification of alternative route options (Butt 2021) and use of simulation to improve decision-making (Belhadi et al., 2021). Finally, some authors focus on specific industries or sectors. Some examples are Chowdhury et al. (2020) and Tirkolaee et al. (2022). The former authors explore mitigation strategies tailored to food and beverage industry, such as First Expiry First Out approach and product rotation. Both strategies can minimize the risk of product expiry that may affect food and beverage companies during a pandemic. Tirkolaee et al. (2022) focus instead on the production and distribution of face masks, proposing a model to develop a sustainable closed-loop supply chain network.

From the previous overview, a relevant gap emerges. Even if many studies deal with the mitigation strategies useful to face the COVID-19 disruptions, the solutions investigated and discussed by scholars are mainly linked to the use of new technologies or the redesign of supply chain structure. None of previous studies looks at how the mitigation process is and has been managed from an organizational point of view, in terms of organizational structure and culture, procedures and coordination mechanisms, steps and sequence of the mitigation process.

Putting the spotlight on the organizational aspects of the mitigation process is important for many reasons:

- First of all, as suggested by Dabhlikar et al. (2016), a successful mitigation process requires firms to clearly understand what actions to take, when, where and how. Even if the literature provides such indications for general disruptions (see Sect. 2.1), they may not be suitable or equally effective in a pandemic setting, which is a unique disruptive event (Belhadi et al. 2021; van Hoek 2021).
- Second, it offers the opportunity to stimulate the debate on organizational structure, a key driver of performance (DeCanio et al. 2000), addressing issues such as how to divide, organize and coordinate the activities during the mitigation process. This may help companies to maximize the effectiveness of mitigation actions, thus minimizing the negative effects of pandemic’s disruptions.
- Third, understanding how to structure the process and how to manage the interfaces across multiple organizations involved in the task could be useful to identify the capabilities and competencies that companies should try to develop, not only during the mitigation process, but also later, to prepare for any future comparable situations.

Accordingly, the aim of this paper is to explore the reactive organizational practices to manage the mitigation process in the context of COVID-19 pandemic. Besides achieving the aforementioned benefits, this analysis allows also to clarify if and how the reactive organizational practices used in a pandemic context differ from those proposed in the disruption management literature.

## Methodology

To address the research questions, we adopted a single case study methodology. Case study research allows to explore in detail the issues of interest and to acquire an in-depth understanding of complex phenomena (Eisenhardt 1989). In addition, it is particularly suited to answer how, what and why questions (Voss et al. 2002; Yin 2014), thus perfectly fitting our case. Single case studies, in particular, despite being less generalizable than multiple case studies and lacking the possibility of making cross-case analyses, have the advantages of exploring more intervening variables and producing extra and better theory (Dyer and Wilkins 1991). Furthermore, they allow to perform a more careful study and identify all the existing mechanisms underlying a phenomenon, including both expected and unexpected ones (Yin 2014). Accordingly, since our aim and research questions are exploratory in nature and require accurate and deep analyses of the organizational aspects of the mitigation process, this solution was considered the most appropriate for our research.

The company selected for the case study, which will be referred to as Company Alfa, is a world-class producer of heating elements for a wide set of applications, that range from heating, refrigeration and conditioning to food, laundry and medical services. The company is structured in two business units, which refer to two distinct geographical areas and present the characteristics reported in Table 3. Both business units produce standardized products and have a flat demand, which does not show any seasonality trends.

**Table 3:** Company Alfa’s data

	Business unit A	Business unit B
Served market	Global	Global
Employees	3,800	3,600
Income	366 M€	285 M€
Production plants	Europe: 1 Asia: 2	North America: 2 South America: 2
Finished products	2,300 pc	12,400 pc

Choosing Company Alfa was based on purposeful sampling, which indicates the selection of an appropriate and rich case from the perspective of the research goal (Patton 1990). In particular, three reasons drove Company Alfa’s selection. First, it is a medium-sized company with a good organizational structure and advanced internal risk management practices. This aspect guaranteed that (1) a sufficiently articulated set of practices and actions was put in place during the mitigation process, and that (2) people with different backgrounds were involved in the mitigation process. In a smaller company, it would probably not be possible to observe the most evolved organizational dynamics of the mitigation process, as this latter would inevitably be simpler and less structured. Second, Company Alfa has a complex and global supply chain, with articulated transportation routes and suppliers and customers located all over the world. This complexity made the company highly exposed to the COVID-19 challenges and required it to coordinate the reactive organizational practices with several partners, thus providing several tips for the mitigation decisions and activities related to the *interfaces* category (see Sect. 2.1). Third, Company Alfa is a successful example of COVID-19 disruption management because, thanks to a structured organizational mitigation process, it was able to avoid production interruptions and minimize the negative effects of the disruptions (see Sect. 4.3). Accordingly, it can be considered an exemplary case study to learn from and to address the research questions.

### Data collection

Data was collected through semi-structured interviews conducted in November 2020-February 2021. The interviews’ content was linked to the mitigation practices implemented by Company Alfa from the beginning of the COVID-19 spreading, namely from January–February 2020. Following the recommendations of Yin (2014), we created a research protocol with open-ended questions to drive the interviews, as shown in Table 4. Besides exploring the challenges faced by Company Alfa during the pandemic, we collected detailed information on the organizational practices implemented to manage them, ensuring that all the aspects concerning *governance*, *interfaces* and *operations* were handled during the interviews. Overall, we carried out 7 interviews with 6 people managing the main business areas of Company Alfa (see Table 5). These people were selected because they were part of the task force created by the company for the COVID-19 disruption management. Before carrying out the interviews, we sent an email to the participants explaining the goals of the research and providing the research protocol. Each interview, conducted using online platforms, had a duration varying between 45 and 90 min. The first interview was carried out with the global supply chain director, who is also the leader of the COVID-19 task force, and it aimed at collecting information on the overall management of the mitigation process. Subsequently, we interviewed the various area managers to deepen the aspects related to their specific business areas. Finally, we carried out a group interview with all the participants to validate the process and the collected information. During the group interview, we also remotely accessed company’s reports and documents (e.g., *disruptions' register*) and collected information on key performance indicators. The questions reported in Table 4 were slightly adapted depending on the interview type and the respondent role; the level of detail of the discussion on the various themes changed accordingly. The interviews, carried out in Italian, were recorded with the interviewees’ permission, transcribed and then translated in English language.

**Table 4:** The interview protocol

Sections	Exemplary questions
Interviewee	• What is your current position in Company Alfa? • How long have you been working in Company Alfa?
Impact of COVID-19	• What challenges/disruptions did or does Company Alfa face because of COVID-19 pandemic? • Can you provide a specific example of challenges faced by your business area?
Actions implemented to face the COVID-19 disruptions	*Governance* • Did your company create a specific organizational structure to face the COVID-19 disruptions? • Who was responsible of the overall mitigation process? • Who was involved? How? • What was your role? • How were final decisions taken (team, individual, other)? *Interfaces* • Were there any interactions between business areas during the mitigation process? • Did your company define any coordination mechanisms with supply chain partners? If yes, which ones? How were they implemented and with whom? *Operations* • How did your company organize the problem-solving process to face the COVID-19 challenges? What steps were carried out and by whom? • Did you introduce any specific procedures to manage the mitigation process? • Can you provide a detailed example of how you (and your colleagues) solved one of the challenges faced by your business area? *Others* • Are there any other organizational aspects concerning Company Alfa’s mitigation process that we did not discuss but may be important for our purposes?

**Table 5:** Overview of interviewed people

Interview type	Respondent role	Duration
Single interview	Global supply chain director	90 min
	Demand manager	45 min
	Operations manager	50 min
	Logistics manager	70 min
	Procurement manager	70 min
	Inventory manager	50 min
Group interview	All of the above figures	90 min

### Data analysis

The data analysis process consisted of two steps. First, we analyzed the data concerning COVID-19 impact, summarizing the related information and identifying the main challenges that affected each functional area. Then, we coded the data concerning the mitigation process, identifying the organizational practices discussed by the respondents and grouping them according to the categories illustrated in Table 1. This activity consisted of three sub-steps and was carried out using a combination of deductive and inductive approaches, following Wholey et al.’s (2010) guidelines. In the first sub-step, a clear definition of *governance*, *interfaces* and *operations* was developed and shared among the three authors. This guaranteed that all of them understood and interpreted the three categories in the same way. In the second sub-step, each author independently read the transcribed interviews and identified text segments (i.e., short paragraphs or sentences) referring to the organizational practices implemented by the company. Each text segment was then coded by each author with one of the three abovementioned categories, relying on the definitions developed in the first sub-step. For instance, when an interviewee referred to “interactions”, “information sharing” or “coordination” activities, the relative text segment was typically coded with the *interfaces* label. The authors then met, compared the results of the coding process and solved the few misalignments. For instance, they agreed that in some sentences, practices belonging to two different categories could be identified. A double label was thus assigned to these text segments. In addition, one particular matter had to be addressed. Since monitoring activities were frequently mentioned during the interviews, one author wondered if it was appropriate to consider a fourth category of practices called *monitoring*. After a brief discussion, the authors decided to avoid this change because the relative practices would be the same of the *operations* category. Indeed, real-time monitoring activities characterize every operational practice carried out in a pandemic context, since they enhance the ability to quickly react to these extreme disruptions. At the end of the second sub-step, all the text segments that were not associated to any category were excluded from further analysis. In the third sub-step, each author independently reviewed the text segments coded within each category and inductively identified a list of implemented practices. A meeting was then organized to compare the three lists of practices developed by the three authors. The seven practices common to all the three lists were immediately confirmed. For all the others, a debated discussion was initiated. The main point of divergence was not whether a practice had been implemented or not. The issue was whether it was worth listing it as a separate practice instead of including it as a specification of one of the other practices. An alignment was found through both a brainstorming among the authors and some additional feedbacks collected from the interviewees. For instance, during the interviews, we were told that a specific document, called *disruptions’ register*, was created to store all the information concerning the mitigation process. According to two authors, this activity should be listed as a separate practice, while the third author considered it as an example of adoption of well-defined procedures (an additional practice already included in all the authors’ lists). The global supply chain director of Company Alfa was thus contacted to provide further information concerning the *disruptions’ register*. From this discussion, it emerged that in fact this document was of key importance for the management of the mitigation process, since it helped the company not only to have an updated overview of the situation, but also to observe the evolution of the mitigation process and of the implemented solutions. Being this aspect quite peculiar, also the third author finally agreed to list it as a separate practice. At the end of the process, a set of ten practices was created by the authors. Furthermore, a table with a list of these practices, together with the related description was created. Indeed, besides identifying and listing the practices implemented in *governance*, *interfaces* and *operations*, it was also important to thoroughly analyze them. This allowed, in a further step, to identify common traits and differences vis-a vis the mitigation practices described in disruption management literature (see Sect. 5).

Reliability and validity of the overall process of analysis were guaranteed following the guidelines of Yin (2014). First, as we already mentioned, we used a pre-defined interview protocol and collected information from six different respondents. This allowed to increase the reliability of the research, the transparency of the process and the validity of the results. For instance, the fact that six different people pointed out the same characteristics of the decision-making process (i.e., team discussion, alternative solutions development and effectiveness monitoring) gave us sufficient confidence that no bias was introduced in the results. Furthermore, we combined data gathered through the interviews with internal documents and publicly available material, thus enabling data source triangulation. In particular, Company Alfa shared with us an excerpt of the *disruptions’ register*, which allowed us to corroborate many details of the implemented practices. In addition, we also analyzed two public interviews, where the CEO of Company Alfa provided some comments on the situation faced by the company during the pandemic. Despite the fewer details, if compared with the content of our interviews, the information provided in these public documents validated the findings on the overall organizational approach adopted by Company Alfa for the mitigation process. Finally, all interviewees agreed to provide feedbacks on the results obtained, allowing the authors to clarify any doubts about the collected data and providing a further support to the validity of the findings. In particular, the interviewees were contacted in two situations: (1) to solve the eventual misalignments in the third sub-step of the coding process (i.e., identification of a list of organizational practices), as we previously described; and (2) to approve the description of the practices included in the final table.

## Case description

### Impact of COVID-19 on Company Alfa

The COVID-19 pandemic impacted the entire Company Alfa’s supply chain.

The first problem concerned the availability of raw materials, whose scarcity progressively increased during the first months of the pandemic. On the one hand, many suppliers closed their activities because of financial or labor scarcity problems. On the other hand, the service level of the remaining suppliers often decreased, and the deliveries agreed with Company Alfa, in terms of both time and quantity, became less reliable.

As concerns the demand side, the problem was not only the unpredictability, but also the extent of variability of customers’ demand. Many customers closed their companies, while some others, whose plant continued to operate, experienced an increase in consumption up to 200%, which was completely unpredictable. This situation made the production planning process increasingly difficult and the inventory stock levels extremely variable.

The main challenges regarded however the logistics activities. The capacity of many freight ship companies saturated in a short time. As a result, the costs of alternative transportation modes (e.g., airways) significantly increased. Furthermore, it became increasingly difficult for road carriers to find available drivers, being these not willing to cross Europe and, in particularly, to move to Italy, which was put under the spotlight by mass-media worldwide because it was among the early countries impacted by the first pandemic wave. Crossing international borders created another tough challenge for Company Alfa’s logistics processes. Many countries closed their borders, creating long queues in custom clearance activities, while others introduced quarantine periods for people coming from abroad regions. In both cases, the direct consequence was a potential explosion of delivery times and the need to identify alternative route options.

However, besides the abovementioned problems, the most critical aspect was that the situation was continuously evolving, since new disruption points emerged every day in different areas of the supply chain and in different locations. In this sense, the toughest challenge concerned again the logistics area. Every day a different country closed its borders and another reopened them. In some cases, the entrance into a country was open but the exit was closed. Even when borders were open, queues of 40–50 km could be created because of restrictions in border controls introduced by some countries.

### Reactive organizational practices implemented by Company Alfa

To face the challenges previously discussed, Company Alfa designed and activated a mitigation process consisting of a set of organizational actions and practices involving all the three categories listed in Table 1.

As regards *governance*, the overall management of the mitigation process was assigned to a cross-functional task force, created by selecting young, flexible and creative managers belonging to the main business areas of the company. The global supply chain director was in charge of leading the task force. As a recognized leader, he was entrusted with the autonomy of taking the financial and organizational decisions deemed most appropriate for solving the identified problems. Furthermore, each member of the task force was required to monitor the situation and collect daily information on his/her own functional area (e.g., customers’ and suppliers’ closures, materials availability, carriers’ available capacity, etc.). Particular attention was given to the overall task force management. The leader rewarded initiatives instead of punishing mistakes and encouraged the development of numerous and quick, even if rough, ideas to solve the problems, instead of slow optimal solutions. For this purpose, a rewarding system assigning appropriate scores to the task force members was created. Every time a member of the group proposed an idea, he/she earned some points, which varied according to the type of problem that the idea would solve. In particular, the leader assigned: 1 point for the ideas concerning a problem related to the stock levels; 2 points for the ideas aimed at avoiding a potential disruption of the production lines; 3 points for those related to a problem potentially affecting the final customers. If the proposed idea effectively solved the problem, this score was doubled. Therefore, even ineffective ideas allowed the task force members to increase their score. The members achieving the highest scores were rewarded with a cash prize, a training course or the involvement into innovative company projects. Finally, emotional moments to share extra work experiences were also organized to favor team building and trust development.

For what concerns the management of *interfaces*, coordination and cooperation inside and outside company’s boundaries were promoted in the mitigation process. Daily meetings were organized among the task force members to share the updated situation of the various functional areas and to support the task force leader in identifying quick and shared solutions for solving the problems and managing the disruptions. Furthermore, constant interactions and information sharing activities with supply chain partners were carried out, not only to develop an updated overview of the supply chain situation, but also to quickly identify new disruption points and mitigate their effects. For instance, the interactions with transportation providers turned out to be fundamental, especially at the beginning of the pandemic, to avoid transportation costs increase and, probably, lead time explosion for the items coming from Asian suppliers. Indeed, in January 2020, when the pandemic was struggling China, the capacity of freight ship companies started decreasing considerably. Thanks to the coordination mechanisms that were put in place, Company Alfa identified this problem early enough and booked a certain number of shipments for the following months, anticipating the related expenses but securing the availability of the maritime carriers. Companies that waited too much to take this decision had to choose alternative transportation modes, the airways in particular, whose costs rose, up to five times. The interactions with truck drivers were instead fundamental to avoid lead time explosions for the intra-European transportations. Drivers continuously informed Company Alfa about the traffic situation, especially at the borders. Thus, when a border closed or a long queue was in place because of borders’ controls, Company Alfa could immediately work to find alternative transportation routes and inform the other carriers directed at the same border.

**Fig. 1:**
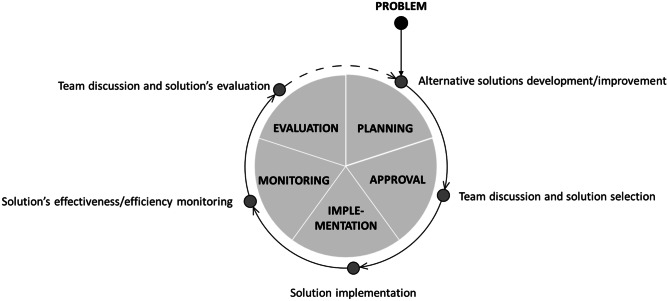
Structure of the problem-solving process in Company Alfa

As far as *operations* is concerned, the overall mitigation process was organized in a very structured way. First, detailed procedures were designed and used to monitor and manage supply chain disruptions as they appeared. The members of the task force constantly updated and shared files with (1) suppliers’ closures, (2) customers’ closures, (3) borders’ situation and (4) average components’ coverage. Furthermore, specific countermeasures were immediately applied to solve the less challenging issues (e.g., orders postponement in case of customers’ closure). For the most severe disruptions (e.g., new borders’ closures, new carrier problems, new transportation route interruptions), a problem-solving process involving the entire task force was instead activated. From the interviews, it emerged that this process had a peculiar structure. Indeed, the severe issues faced by Company Alfa were unprecedented, there were no pre-defined solutions to manage them and there was no time to look for optimal countermeasures. Therefore, the company adopted a trial-and-error approach based on continuous improvement, as highlighted in Fig. 1. Once a new problem or disruption was identified, the task force members met together and brainstormed to develop as many ideas as possible in the shortest time possible. The best ideas were immediately tested, their effectiveness monitored, and, in case of positive results, these ideas were directly implemented and improved along the way. This guaranteed proper flexibility and reactivity to mitigate the effects of the new problems arising every day. A *disruptions’ register* was also created to store information about the disruptions, the proposed solutions, their positive or negative effectiveness and eventual ideas for their further improvement. As Fig. 1 shows, team discussion, alternative solutions implementation and effectiveness monitoring were key aspects of the overall process, which was based on a cyclic *learning-by-doing* approach. However, from the interviews with the task force members, it also emerged that, while at the beginning of the pandemic the uncertainty was extremely high and the errors several, with the passing of time the task force members started learning from previous disruptions and developed new knowledge and capabilities to face them. Thus, the problem-solving process became more and more linear and lean, bypassing not only the cyclic approach, but also many previously implemented steps (e.g., team discussion and effectiveness monitoring).

A summary of all the organizational practices described above is provided in Table 6.

**Table 6:** Reactive organizational practices implemented by Company Alfa

Categories	Implemented practices
*Governance*	• Creation of a cross-functional task force of young, flexible and creative managers representing the main business areas • Identification of the task force leader • Assignment of clear roles and responsibilities • Implementation of team building activities • Establishment of a system rewarding innovative ideas and initiatives
*Interfaces*	• Scheduling of daily meetings among the task force members • Development of coordination mechanisms with supply chain partners based on information sharing
*Operations*	• Adoption of well-defined procedures for demand management, resource reallocation and production adaption • Development of a cyclic problem-solving process with a trial-and-error approach that encourages quick and rough solutions rather than slow and optimal ones • Creation of the *disruptions’ register*

### Results achieved by Company Alfa during the COVID-19 pandemic

**Fig. 2:**
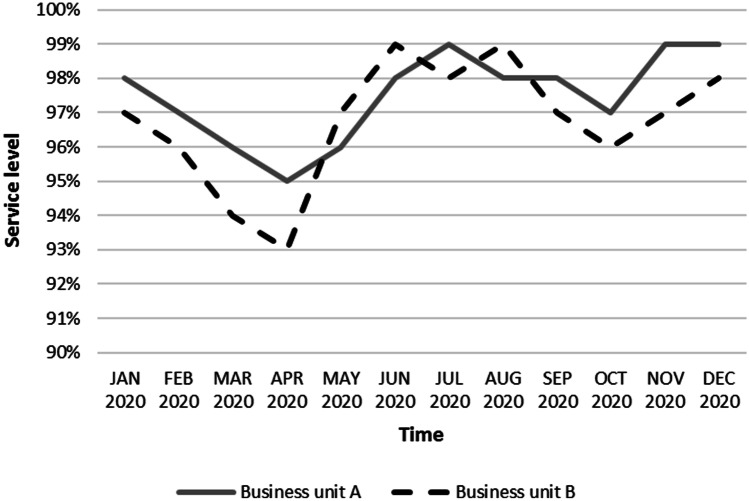
Market service level trend in Company Alfa during 2020

**Fig. 3:**
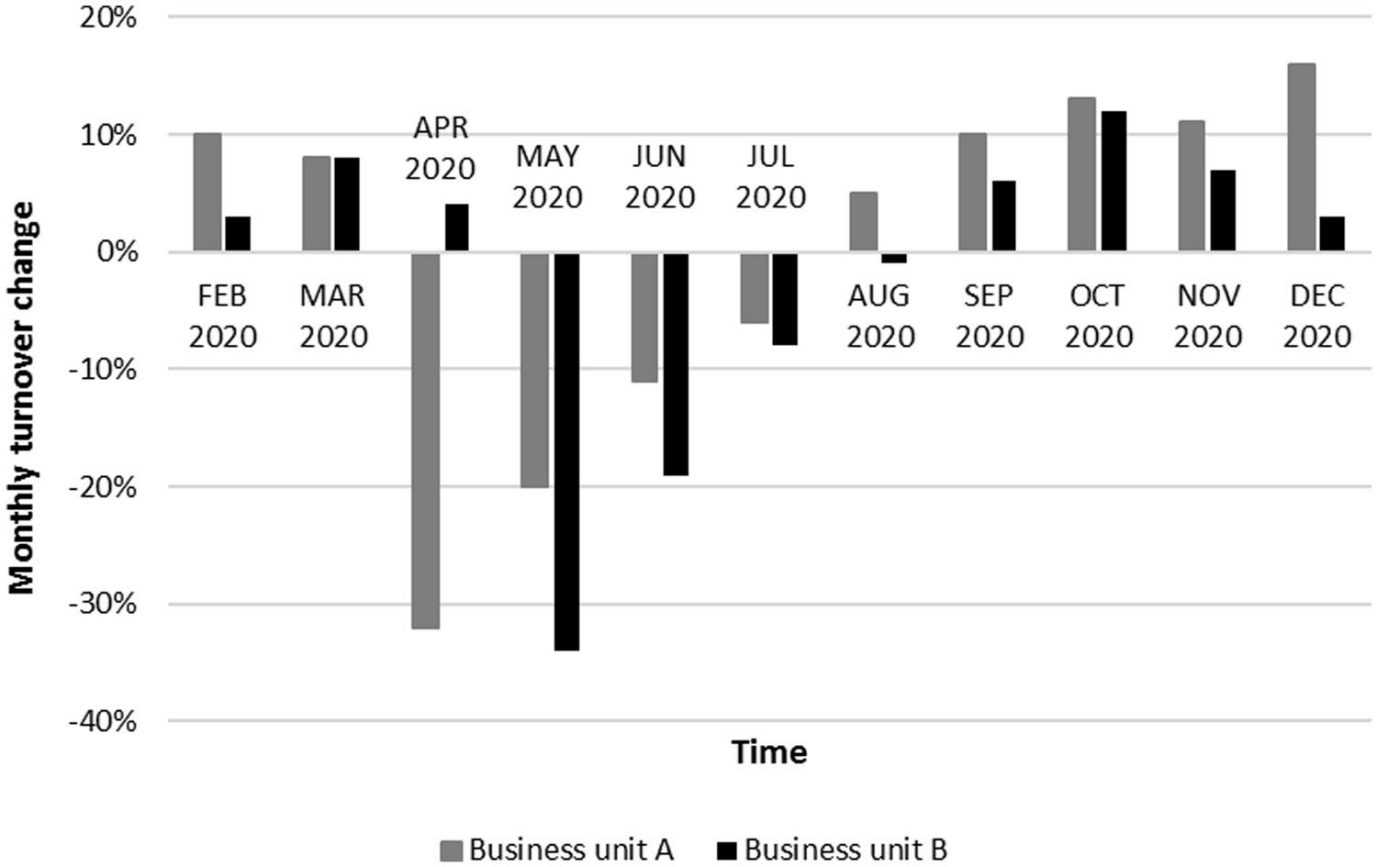
Turnover trend (%) in Company Alfa (reference: January 2020). The turnover of January 2020 was taken as a point of reference for the turnover change analysis as it represents a standard value that, in absence of the pandemic, would have been repeated almost constantly throughout 2020. As we explained in Sect. 3, Company Alfa’s demand is indeed quite flat; thus, all the positive/negative changes depicted in the figure are due to the pandemic effects

Thanks to the well-structured mitigation process, Company Alfa could reduce the negative effects of the COVID-19 disruptions. In particular, it was able to guarantee a very high service level to customers during all the worst months of the pandemic, as shown in Fig. 2. Even if during these months many customers of Company Alfa stopped their production, guaranteeing a good service level to those still in operation was not an easy task. First, there was a scarcity of raw materials and an overall reduction of the suppliers’ service level, which made it more difficult for Company Alfa to comply with its production plan. Second, the distribution activities were often challenged by the unavailability of transportation providers and the constraints applied to custom clearance activities, creating additional obstacles for the on-time delivery to Company Alfa’s customers. Third, customer demand became unpredictable since the mix and volumes of products required by customers varied significantly. The result shown in Fig. 2 could thus be achieved only thanks to a proper management of all the disruptions caused by the pandemic to the various business areas. Obviously, the company experienced some losses because of the pandemic, as testified by Fig. 3, which shows how the turnover of Company Alfa varied in the 2020 months compared to January 2020 (when the market was not affected by the pandemic yet). It is evident that from April to July 2020 the monthly turnover significantly decreased, thus it is plausible to argue that the turnover reduction positively affected the service level. However, it should be noted that the service level remained always high, even in the months of rising turnover.

**Fig. 4:**
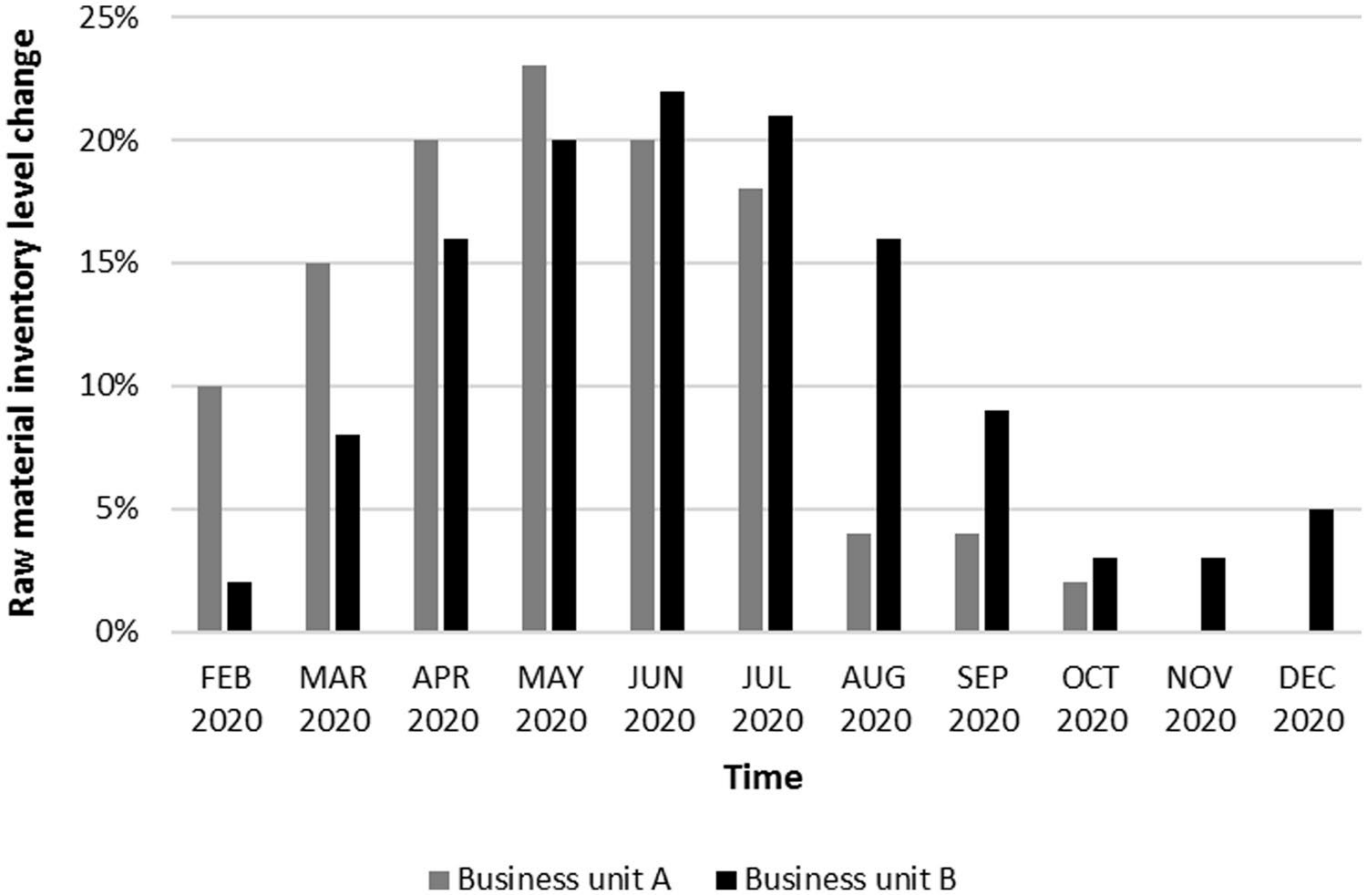
Raw material inventory trend (%) in Company Alfa (reference: January 2020)

It is also worth highlighting that the average stock levels of the company increased quite significantly during the pandemic. Figure 4, which presents the percentage change in stock compared to January 2020, clearly shows that, in the central months of the year, increases in inventory levels have on average reached (and sometimes exceeded) the value of + 20% compared to January’s stock level. Despite the high maintenance cost that these quantities entail, the situation gives further support to the effectiveness of Company Alfa in managing the disruptions of the supplier network. The ability to procure all the materials needed for the production allowed Company Alfa to ensure the continuity of supply to all the customers. The anticipation stock the company was able to create in the worst phase of the pandemic determined its ability to chase the increasing demand in the second part of 2020. Thanks to material availability, Company Alfa was also able to accept “last minute”, unplanned customer requests, thus increasing customer satisfaction. This result improved the company’s reputation and image and consequently allowed it to strengthen its current and future position on the market. Many competitors of Company Alfa were indeed not able to properly face the pandemic disruptions and lost a significant part of their market shares, which was gained by Company Alfa. We can thus conclude that the *price paid* to obtain all these results was completely offset by the earned benefits.

## Analysis

By comparing the reactive organizational practices implemented in Company Alfa (Table 6) with those discussed in the disruption management literature (Table 1), it is evident that many common traits do exist. As concerns the *governance*, the creation of a cross-functional recovery team, the identification of an appropriate leader for the team, the clear definition of roles and responsibilities, which have a key importance in general disruption management, represent a fundamental starting point also for the mitigation process of a worldwide pandemic. A similar conclusion can be drawn for the other categories (i.e., *interfaces* and *operations*), where the crucial importance of coordination and cooperation inside and outside company’s boundaries, as well as the use of structured information sharing process are confirmed.

A general conclusion from the above is that the organizational mitigation process of a pandemic seems to be quite similar to that of general disruptions. However, from a deeper reflection on the case study it also emerges that some differences exist, mainly concerning how the practices (rather than what practices) are implemented.

As far as *governance* is concerned, the empirical evidence shows the importance of developing a decentralized decision-making to quickly react to the daily disruptions. The task force and the leader had the autonomy of implementing and testing the countermeasures deemed most appropriate to solve the problems and this allowed to bypass top management involvement that can often slow down the decision-making process. In this sense, the case study seems to suggest that the sense of urge due to limited time available to mitigate the pandemic effects requires a capable and empowered task force, being top management support alone insufficient to properly organize a quick and effective mitigation process. A further peculiar aspect worth discussing in the *governance* category is the environment that companies should create to maximize the effectiveness of the mitigation process during a pandemic, in terms of culture and mindset. According to the case study results, a successful element of Company Alfa’s mitigation process was the overall task force management, based on the promotion of collaborative culture and trust development. This was effectively integrated with the creation of extra-work experiences to favour team building. Furthermore, the executives and the task force leader worked to develop a risk-taker mindset among the task force by adopting an incentive system that promoted the development of innovative and quick solutions. All the interviewees told us that, when a new issue arose, they were encouraged to develop numerous ideas in the shortest time possible and they were rewarded accordingly, no matter of the ideas’ effectiveness. In addition, errors and mistakes were not punished, providing a further incentive to exploit employees’ creativity and improvisation qualities. It is worth highlighting that this approach may also have some drawbacks. The frequent meetings and interactions within the task force, if not well managed, may become ineffective and dispersive. Furthermore, the development of many alternative solutions may be linked to the loss of best ideas in the noise. Therefore, the approach adopted by Company Alfa, to be effective, required a clear definition of the goals to be achieved in each meeting, but also a leader who properly managed these meetings, avoiding the proliferation of discussions not strictly related to the mitigation process.

As regards the *interfaces*, a peculiar aspect of the pandemic setting concerns the final purpose of coordination and information sharing with partners. Unlike conventional disruptions, these activities seem to be important not only to carry out the mitigation actions and processes, but also to quickly identify the new disruption points or events that emerge every day, as well as to forecast them in advance. This was particularly evident for the transportation activities between European countries, where governments continuously changed the regulations on logistics and freight transport and it was thus necessary to listen to weak signals (e.g., monitoring press agencies), to be in continuous contact and to coordinate with supply chain partners to avoid an explosion of delivery times. Therefore, the disruption identification phase, that typically precedes the mitigation process in general disruption management, should be considered as a part of the mitigation process itself in case of epidemic outbreaks, hence increasing the need of external coordination and information sharing.

For what concerns instead the *operations* category, interesting elements of peculiarity of the pandemic mitigation process concern the structure and the characteristics of the problem-solving process. First and foremost, empirical evidence shows that, especially at the beginning of the epidemic outbreak, the systematic problem-solving process was cyclic rather than linear, as depicted in Fig. 1. The basic ideas are: (1) working in a cross-functional team to develop potential solutions that are not necessarily excellent but obtained quickly, (2) testing them immediately, (3) monitoring and verifying their effectiveness within the team and, in case of positive results, (4) implementing them on a large scale, continuously looking for potential improvements to be applied along the way or in other contexts. We highlight that, even if Company Alfa did not fully exploit them, the new technologies could significantly speed up this process; for instance, they could be used to simulate the solutions' effectiveness before their practical implementation, in particular for logistics problems. The use of technology appears thus to be an important, although not fundamental, factor for the management of the pandemic's mitigation process. Anyhow, the cyclic problem-solving process adopted by the company reveals that there are no pre-defined solutions to face a new pandemic and the short time available for the reaction does not allow to look for optimal solutions nor to prepare in advance a completely pre-defined mitigation process. A trial-and-error approach, based on a critical evaluation and monitoring of the developed and tested ideas, seems instead the most appropriate way to organize the problem-solving process. In this sense, every error is an opportunity to benefit from experimentation, learn and consequently adapt future decisions (i.e., *learning-by-doing* approach). This also explains why cycle after cycle experiences cumulate and the process becomes more and more linear and lean: thanks to the evaluation and monitoring of the various solutions and of their effectiveness, the task force progressively acquires appropriate knowledge and experience, speeding up the overall problem-solving process. Not by chance, Company Alfa decided to keep some practices implemented during the pandemic's mitigation process also in the future, in order to strengthen the learning process and experience of the workforce. For instance, it established the organization of regular meetings within the pandemic’s task force to reflect on the weakest points of the company’s supply network and become more and more aware of the potential disruptions. In this way, Company Alfa expects to be better prepared in case of future extreme events and to re-implement all the aforementioned strategies with even stronger effectiveness.

**Fig. 5:**
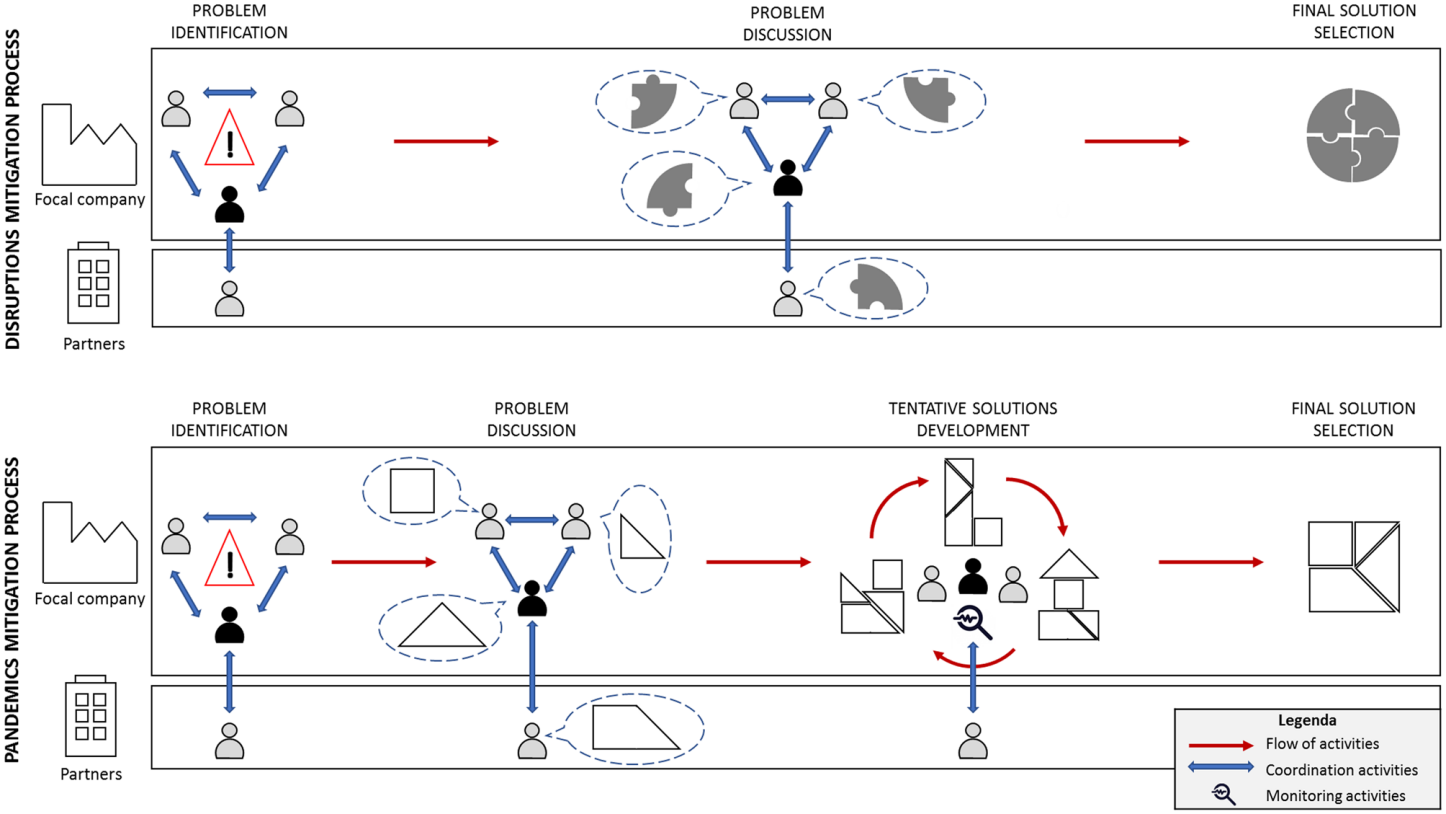
A comparison between disruptions and pandemics mitigation processes

To sum up, Fig. 5 provides a graphical comparison between disruptions and pandemics mitigation processes, summarizing the key points emerged from the previous analysis. In both situations, companies should create a task force managed by a leader (i.e., grey and black stickmen) and develop coordination mechanisms within and outside the organization (i.e., blue bidirectional arrows). However, while in disruptions management the problem discussion can be quickly directed towards the identification of a solution thanks to the contributions (i.e., puzzle pieces) provided by each task force member and, eventually, by external partners, in a pandemic setting the situation is much more complex. The contributions provided by the single members (i.e., geometric figures) can be combined in many different ways, generating different potential solutions. The problem discussion activity should thus be followed by a cyclic process of tentative solutions development, where the task force members test and monitor (i.e., magnifying glass) the different options, till the final optimal solution is found. Creativity, improvisation and risk-taking are thus key elements to effectively manage the cyclic problem-solving process of an epidemic outbreak.

## Discussion

This research provides the following theoretical contributions.

The first contribution, related to RQ1, concerns the evidence that the overall management of pandemic’s organizational aspects is not particularly different from that proposed in the disruption management literature. The importance of many practices recommended by previous studies on disruption management (e.g., Norrman and Jansson 2004; Craighead et al. 2007; Macdonald and Corsi 2013; Dabhilkar et al. 2016), such as the creation of a cross-functional task force with precise roles and responsibilities, the implementation of clear procedures, the scheduling of daily meetings among the task force and the implementation of coordination mechanisms inside and outside companies’ boundaries, is thus confirmed also for a pandemic setting. Furthermore, this study confirms that, for an effective pandemics’ mitigation process, a concurrent investment is needed in *governance*, *interfaces* and *operations* categories, as claimed by Norrman and Jansson (2004), Scholten et al. (2014), Dabhilkar et al. (2016) and Chen et al. (2019) for general disruptions.

However, this study also reveals some peculiarities that differentiate the pandemic’s mitigation process from that of general disruptions and represent an original theoretical contribution related to RQ2. Indeed, even if the general guidelines provided by the disruption management literature are valid also in a pandemic setting, some differences on how the conventional practices should be implemented in this latter context emerge, especially in *governance* and *operations* categories. First of all, empirical evidence shows that the effectiveness of the task force and the engagement of the related members can be enhanced through team building activities. This concept is not new and the literature extensively discusses the benefits of team building activities, especially for project management (e.g., Thomas et al. 2008; Pollak and Matous 2019). However, in the context of disruption management, scholars do not give particular attention to this aspect, probably because the recovery team is built ad hoc to solve a specific problem and it therefore operates for a limited time period. In contrast, the mitigation process of a pandemic such as the COVID-19 is much longer, demanding and stressing for the team members, continuously urged to intense trouble shooting. A second peculiar trait of a pandemic’s mitigation process emerged from the empirical study is the development of a risk-taker mindset. This aspect can be discussed in light of the regulatory focus theory, according to which two attitudes towards the risky decision-making behavior exist: the *promotion focus* behavior, which looks at the achievement of rewards, and the *prevention focus* one, which looks at the avoidance of punishment (Higgins 1997). People having a *promotion focus* behavior use more creative problem-solving skills, are more willing to take risks and tend to prefer innovations to the status quo maintenance (Kark and Van Dijk 2007; Cantor et al. 2014). These three characteristics are exactly what Company Alfa searched for the mitigation process and the incentive system, the attention on team building and the managerial attitude coherently converged towards this goal. Thus, the present research suggests the need to adopt a *promotion focus* behavior among the task force during the mitigation process of a pandemic, an aspect that does not emerge in the disruption management literature. Finally, a further relevant aspect worth discussing is the overall structure of the pandemic’s mitigation process. This research suggests that, especially at the beginning of the epidemic outbreak, the systematic problem-solving process recommended by many scholars (e.g., Dabhilkar et al. 2016; Chen et al. 2019) should be cyclic rather than linear. While the linear implementation of pre-defined procedures, protocols and solutions seems to be enough to effectively mitigate the challenges imposed by general disruptions (see e.g., Norrman and Jansson 2004; Tukamuhabwa et al. 2017), this approach is not suitable for a pandemic setting. In this context, companies need to develop completely new solutions, addressing unprecedented challenges and thus improving the process along the way. Furthermore, if the disruption management literature recommends to analyze past disruptive events and learn from them (e.g., Scholten et al. 2014), this research highlights the importance of learning from actual experiences and current errors. Therefore, companies must be aware that creativity and improvisation are at least as important as capacity and experience for an effective pandemic’s mitigation process.

Besides theoretical contributions, this research has also relevant practical implications that allow to identify original suggestions and guidelines to manage the organizational aspects of a pandemic’s mitigation process.

First, the evidence shows that it is fundamental to develop a proper organizational structure by creating a cross-functional task force managed by a capable leader and composed of young, creative and flexible people. This task force should be supported by an incentive system and managerial attitude that support a risk-taker mindset, team building and trust development. During a pandemic there is no time to wait for optimal or no-risky solutions. Probably, these latter do not even exist. It becomes thus fundamental to create an environment that favours a *promotion focus* behaviour, rather than a *prevention focus* one.

Second, this research highlights the importance of monitoring existing and forecast potential disruptive points. During a worldwide pandemic, contrary to what happens in traditional disruptions, troubles and failures are not limited to a specific portion of the supply chain nor to a unique point in time. This means that the disruption identification phase does not completely precede the mitigation process but becomes a sub-phase of this latter. Companies should thus share information and coordinate the activities with partners not only for disruption management, but also for the early identification of new disruptions. The collection of information on countries’ and governments’ new regulations is also a key activity to carry out in the mitigation process.

Third, this research recommends the development of a systematic problem-solving process based on recursive cycles and a consequent change of mindset. In the highly uncertainty that characterizes a pandemic, there are no pre-defined solutions to solve the problems and face the disruptions and both strategies and capabilities can be built only *by doing*. Therefore, it is advisable to avoid hampering the teamwork with excessive procedures and bureaucracy, promoting instead a *learning-by-doing* approach, based on continuous improvement efforts.

Fourth, the evidence indicates that reactivity and speed are key elements of the pandemic’s mitigation process. A rough idea implemented early is better than an optimal solution executed later. Quick and effective reactions can be obtained by developing a decentralized decision-making, directing the rewards towards reactivity and flexibility, avoiding the penalization of errors, developing critical thinking skills among the workforce and creating an organizational structure where everyone knows what needs to be done by whom, when and why.

A summary of all the aforementioned guidelines, which represent the main outcomes of this research, is offered in Table 7. We highlight that, besides pandemics, the organizational practices shown in the table can be particularly useful also in the volatile, uncertain, complex, and ambiguous (VUCA) environments that increasingly characterize the current business scenario. They indeed perfectly support the needs of developing agility, sharing information and experimenting new solutions claimed by Bennett and Lemoine (2014) to face the VUCA environments.

**Table 7:** Summary of guidelines to manage the pandemic mitigation process

General guidelines	Best practices and enablers
Develop a proper organizational structure	• Cross-functional task force • Team building activities • Decentralized decision-making
Monitor existing and forecast potential disruptive points	• Information sharing • Activities coordination
Develop a cyclic problem-solving process	• *Learning-by-doing* approach • Creativity skills • Improvisation skills • Critical thinking skills
Change mindset	• Risk-taker mindset • Promotion focus behavior • Creative problem-solving • Incentive system aimed at reactivity and flexibility

## Conclusions

This paper deals with the mitigation process of extreme disruptions in the context of COVID-19 pandemic. Even if the COVID-19 literature is already quite developed, previous studies investigate mitigation strategies mainly linked to the use of technology and the redesign of supply network structure, overlooking the investigation of the mitigation process from an organizational point of view. We address this relevant gap through a detailed case study of an Italian company that, thanks to appropriate organizational practices and strategies, was able to successfully deal with all the disruptions caused by the COVID-19 pandemic. The results show the importance of many reactive organizational practices recommended in the literature for more conventional disruptions, such as the creation of a cross-functional task force, the clear definition of roles and responsibilities and the development of cooperation activities inside and outside companies’ boundaries. In this sense, the overall management of a pandemic’s mitigation process does not significantly differ from that of conventional disruptions. However, some differences on how these organizational practices should be implemented in a pandemic setting emerge from our analysis. Some examples are the implementation of a cyclic rather than linear problem-solving process, the adoption of a *learning-by-doing* approach and the need of a risk-taker mindset. Based on this knowledge, the present study also provides some hints on the main capabilities and strategic behaviors that seem to be required in a pandemic’s mitigation process. Activities such as team discussion and inter-company coordination suggest the importance of having a workforce with team-working and interpersonal communication skills. In looking for potential solutions to face the pandemic’s disruptions, task force members should indeed be able to effectively share their knowledge with others and be open to accept new ideas and perspectives. The occurrence of continuous disruptions and the absence of pre-defined solutions to manage them require instead problem-solving skills, combined with creativity and improvisation qualities. A complete change of mindset, where logic and imagination are combined, is often needed to face the unprecedented supply chain disruptions. These aspects are not currently addressed in the COVID-19 literature and therefore provide relevant theoretical and managerial contributions.

The research contributions should however be viewed in light of some limitations, which further suggest fruitful lines for future research. First, this study relies on a single case study whose results may be influenced by specific environmental and organizational conditions. The location of Company Alfa is certainly an important aspect that may have affected the mitigation process structure, especially if we consider that the first pandemic wave hit the various countries with different timing and intensity. Additional limitations may be linked to the sector and size of Company Alfa, but also to the type of components produced by the company (i.e., standardized products that do not require any customization activities). In this sense, some aspects of the disruption management process were probably simplified since products could be easily shifted among customers. Considering all these peculiarities of Company Alfa, we invite future research studies to corroborate or complement our results by extending the analysis to companies operating in other sectors or countries, having different sizes and managing more complex product personalization activities.

Second, we explored the organizational practices implemented by Company Alfa in the *interface* category considering its viewpoint only. Future research could thus extend the study by including some interviews with all the partners involved in the mitigation process and shifting the focus on the external coordination mechanisms. This would allow to shed light on the drivers, dynamics and barriers of inter-firm coordination during a pandemic’s mitigation process.

Third, this study does not take into consideration the role of environmental and social practices in the mitigation process, despite their relevance in the current business scenario. Since many authors claim that companies with good values of environmental and social performance experienced fewer negative effects during the COVID-19 pandemic (e.g., Hwang et al. 2021), future research could explore if and how these performance outcomes affect the effectiveness of the organizational practices proposed in Table 7.

A further limitation of this research may be linked to the temporal distance between our interviews and the actual execution of the investigated activities. Since the first decisions concerning the mitigation process had to be taken starting from January 2020, the description provided by the interviewees may have been more rational and less impulsive than what would have happened some months before. The results of the empirical study should thus be interpreted with such awareness.

Finally, this study focuses only on organizational reactive strategies to manage the mitigation process of a pandemic. On the one hand, it can be extended by including in the analyses the non-organizational mitigation strategies, such as those explored in Sect. 2.2. This would allow to understand how the importance of different organizational practices varies as the strategies implemented at the technological and network structure level vary. Alternatively, future studies could explore the proper combination between organizational proactive and organizational reactive strategies for pandemic disruptions. In particular, it would be useful for both academics and practitioners to understand if specific reactive strategies or practices are effective only when proper proactive actions have been carried out.
